# Sustainable synthesis of antibacterial 3-aryl-2*H*-benzo[b,1,4]oxazin-2-ones via S_N_Ar Csp^2^–Csp^2^ coupling

**DOI:** 10.3389/fchem.2024.1472342

**Published:** 2024-11-25

**Authors:** Fatemeh Salehzadeh, Maryam Esmkhani, Milad Noori, Shahrzad Javanshir, Aida Iraji, Mohammad Mahdavi

**Affiliations:** ^1^ Pharmaceutical and Heterocyclic Compounds Research Laboratory, Department of Chemistry, Iran University of Science and Technology, Tehran, Iran; ^2^ Stem Cells Technology Research Center, Shiraz University of Medical Sciences, Shiraz, Iran; ^3^ Department of Persian Medicine, Research Center for Traditional Medicine and History of Medicine, School of Medicine, Shiraz University of Medical Sciences, Shiraz, Iran; ^4^ Endocrinology and Metabolism Research Center, Endocrinology and Metabolism Clinical Sciences Institute, Tehran University of Medical Sciences, Tehran, Iran

**Keywords:** Csp 2–Csp 2 cross-coupling reactions, microwave-assisted organic synthesis, nucleophilic aromatic substitution, antimicrobial, natural product cephalandole A

## Abstract

**Introduction:**

The increasing prevalence of antibiotic-resistant pathogens necessitates the urgent development of new antibacterial agents. Concurrently, synthetic chemistry is moving towards more sustainable practices that minimize environmental impact. This study aims to synthesize 3-aryl-2*H*-benzo[b][1,4]oxazin-2-one derivatives, including the natural product cephalandole A, using a sustainable approach that avoids metal catalysts.

**Methods:**

We employed nucleophilic aromatic substitution (SNAr) under microwave-assisted conditions to facilitate the synthesis of the targeted compounds. This metal-free carbon–carbon coupling reaction was optimized for efficiency, yielding good results with reduced reaction times. The synthesized derivatives were then subjected to an *in silico* molecular docking study to predict their antibacterial potential against key bacterial targets, focusing on the binding affinity and interaction profiles.

**Results:**

The microwave-assisted SNAr method provided good yields of 55% to 82% and significantly reduced reaction times ranging from 7 to 12 minutes, simplifying the overall workup process. Among the synthesized compounds, 3-(*1H*-indol-3-yl)-6-methyl-2H-benzo[b][1,4]oxazin-2-one (**6b**) emerged as a promising candidate, demonstrating favorable binding interactions in the molecular docking studies.

**Discussion:**

The integration of sustainable synthetic methodologies with in silico screening offers a novel and effective strategy for drug discovery. Our findings highlight the potential of the synthesized compounds as antibacterial agents and emphasize the importance of adopting eco-friendly approaches in pharmaceutical chemistry. This research contributes to the global effort to combat antibiotic resistance by providing new compounds for further biological evaluation.

## 1 Introduction

Bacterial antibiotic resistance is a critical global health concern that poses a significant threat to human society. This phenomenon occurs when bacteria develop mechanisms to withstand the effects of antibiotics, rendering these medications less or entirely ineffective (Uddin et al., 2021). Two noteworthy bacterial species, *Mycobacterium tuberculosis* and *Streptococcus dysgalactiae*, have garnered particular attention due to their growing antibiotic resistance. *Mycobacterium tuberculosis* is the causative agent of tuberculosis, a devastating infectious disease that primarily affects the lungs but can also affect other organs. Tuberculosis has been a major global health problem for centuries, and the emergence of antibiotic resistance in *M. tuberculosis* strains has compounded the challenges associated with its control and treatment (Batt et al., 2020). Similarly, *S. dysgalactiae* is a bacterial species that can cause a range of infections in humans, including skin and soft tissue infections, invasive bloodstream infections, and streptococcal toxic shock syndrome. These strains resist the most potent and commonly used antibiotics, such as isoniazid and rifampicin, making treatment considerably more complex, costly, and less effective (Commons et al., 2014; Ciszewski et al., 2016; Nevanlinna et al., 2023). Antibiotic resistance in *S. dysgalactiae* is particularly concerning for antibiotics like beta-lactams (e.g., penicillin) and macrolides. Resistance mechanisms in these bacteria may involve the production of beta-lactamases, enzymes that break down beta-lactam antibiotics, rendering them ineffective (Tang et al., 2023; Mathavan et al., 2024).

**FIGURE 1:**
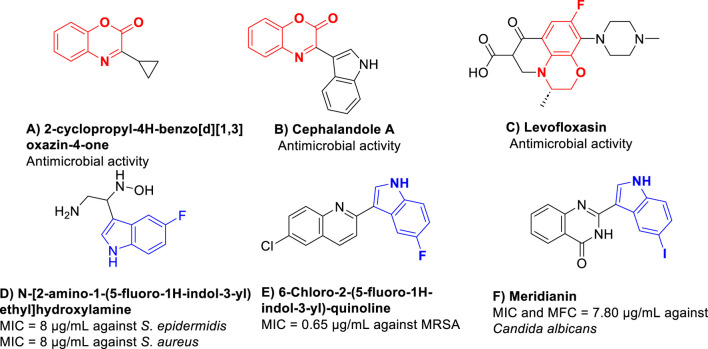
Illustrates a selection of representative benzoxazine pharmacophores (compounds A–F) known for their antimicrobial properties. (Hoemann et al., 2000; Singh and Thakur, 2014; Wang et al., 2019; Qin et al., 2020; Nieto and Lupton, 2021; Zhao et al., 2023).

Given the escalating threat of antibiotic resistance, the development of new antimicrobial agents is essential to combat resistant strains, prevent the spread of untreatable infections, and in global health protection (Miethke et al., 2021). Benzoxazines, as versatile heterocyclic compounds, have emerged as foundational components in synthesizing pharmaceutical compounds. The ability to modify the benzoxazine structure allows for the design of new compounds with tailored properties for specific therapeutic applications, making them invaluable in the development of new synthetic drugs. The ability to modify the benzoxazine structure allows for the design of new compounds with tailored properties for specific therapeutic applications. This versatility is crucial in the development of new synthetic drugs. Benzoxazine derivatives have been evaluated for their potential as drug leads. Modification of the benzoxazine scaffold can lead to improved potency and selectivity for specific biological targets (Tang et al., 2023; Mathavan et al., 2024). Different derivatives were developed as antimicrobial agents, including quinoline, thiazole^7^, chalcone^8^, naphthoquinone^9^, and fluorouracil (Patil et al., 2023). Benzoxazines, as versatile heterocyclic compounds, play a pivotal role as foundational components in synthesizing pharmaceutical compounds. They are prevalent in natural products and biologically active molecules, exemplified by their presence as antibacterial (Mitscher et al., 1987), anticancer (López-Iglesias et al., 2015), antifungal (Waisser et al., 2002), antibiotic (Kushwaha et al., 2020), antinociceptive agents (Zumbrägel et al., 2018), and others. Figure 1 illustrates a selection of representative benzoxazine pharmacophores (compounds **A–C**) known for their antimicrobial properties.

In recent years, there has been a growing body of research works on biologically active compounds with potential as antibacterial agents. Notably, indole-based derivatives (compounds **D–F**) have garnered significant attention for their remarkable efficacy as antimicrobial agents, as evidenced by numerous studies (Hoemann et al., 2000; Burchak et al., 2011; Wang et al., 2019; Qin et al., 2020; Nieto and Lupton, 2021).

The importance of drug discovery cannot be overstated, particularly in the context of developing new therapies to address global health challenges like antibiotic resistance. Traditional drug development processes can take several years, consuming significant time, money, and resources. In contrast, *in silico* drug design has emerged as a revolutionary approach capable of reducing the timeline of drug discovery from years to just days or months. This method not only conserves financial resources and energy but also significantly enhances the efficiency and accuracy of drug development (Boyd et al., 2021; Roney and Mohd Aluwi, 2024).

*In silico* drug design offers a powerful toolset to predict and optimize the pharmacological profiles of potential drug candidates. It enables the rapid screening of large libraries of compounds, identifying those with the most promising biological activity. This approach has been particularly impactful in the field of antimicrobial drug discovery, where it has facilitated the emergence of novel drug candidates designed to target resistant microbial strains (Jabalia et al., 2021; Chang et al., 2022). The ability to predict molecular interactions, binding affinities, and potential off-target effects allows *in silico* methods to rationalize the drug development pipeline and reduce the risk of late-stage failures. Ultimately, *in silico* drug design accelerates the introduction of effective antimicrobial agents to the market, making it a proven, innovative, and compelling approach to modern drug discovery (Zhang et al., 2022; Canales et al., 2024).

In this context, the convergence of benzoxazine and indole structural motifs represents a compelling starting point for developing novel and straightforward synthetic protocols. Such an endeavor has the potential to substantially contribute to the field of synthetic chemistry (Mason et al., 2008). Developing an efficient method to form C–C bonds *via* the arylation of C (sp^2^)–H bonds is one of the important and perpetual subjects in organic synthetic chemistry. One significant category of C–C bond formation processes involves the established transition metal-catalyzed C-arylation through the activation of the C-X bond (where X = I, Br, Cl, and F) in haloarenes. However, this method is plagued by drawbacks such as harsh reaction conditions (high temperature and additives), and the use of costly reagents, diminishing its practicality. Consequently, a sustainable approach was required to address such challenges (Docherty et al., 2023). In recent years, many efforts have been made to synthesize these frameworks, including photo-redox catalysts (Akula et al., 2018; Rostoll-Berenguer et al., 2018), ultrasound techniques (Sharma et al., 2018), and transition metal catalysts (Pandit et al., 2017). Radical oxidation in the presence of hypervalent iodine has recently been reported to synthesize these frameworks (Long et al., 2021). Nevertheless, despite these advances, an efficient, green, and convenient protocol for synthesizing these systems remains a topic of high interest in chemical research (Gräßle et al., 2016).

Nucleophilic aromatic substitution (S_N_Ar) is frequently used in synthetic processes involving halogenated aromatics because halogens are an excellent leaving group. However, the reactions often require harsh conditions and expensive reactants such as transition metal catalysts, which makes scientists hesitant to use them in drug synthesis programs due to the cost and environmental impacts involved (Chu et al., 2011). Microwave-assisted organic synthesis (MAOS) has become one of the most powerful and environmentally friendly tools in synthetic chemistry because of its good features, including reducing the reaction time, energy, and side products and increasing the reaction yields (Rathi et al., 2015). It can easily catalyze reactions such as cross-couplings that need more power (Zhang et al., 2020). MAOS is a valuable tool for synthesizing molecules that require challenging, time-consuming, and low-yield methods. Although several methodologies for synthesizing indole alkaloids have been developed, there are no reports for direct, catalyst-free, and microwave-assisted coupling of 3-chloro-1,4-benzoxazin-2-ones with indoles. Previous pathways have utilized one or two of the items mentioned above. Prompted by the aforementioned topics, we present a simple catalyst-free method to prepare the benzoxazine-containing skeleton through microwave irradiation. We also evaluate the potential of these derivatives as antimicrobial agents against *Mycobacterium tuberculosis* and *Streptococcus dysgalactiae*.

## 2 Results and discussion

### 2.1 Chemistry

**Scheme 1:**
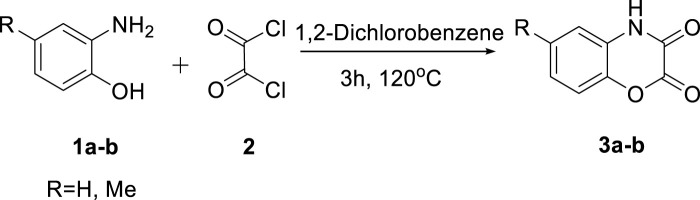
Preparation of 1,4-benzoxazinediones (**3a-b)** ^27^.

The desired precursors 1,4-benzoxazinediones (**3a-b)** were synthesized using a cyclization method that has been previously reported, between 2-aminophenol and oxalyl chloride (Scheme 1) Dickoré et al. (1970). To synthesize the targeted product (**6a)** from the addition–elimination reaction, the reaction conditions were optimized and are tabulated in Table 1. In this regard, 3-chlorobenzoxazine-2-one **(4a)** and indole were exposed to various solvents in the reflux (Table 1, entries 1–5), and the highest reaction yield was found in THF. Hence, the effect of the other conditions, including microwave irradiation, ball-milling, and temperature was examined in THF. Over the years, working on the use of AlCl_3_ as an efficient and inexpensive reagent for the C–C bond forming reactions between heteroaryl chlorides containing–C(Cl) = N–moiety and various arenes or heteroarenes (Kumar et al., 2012), we also investigated the modal reaction in the presence of AlCl_3_ in dichloromethane (Table 1, entry 6); however, the expected product was obtained in low yields. Entry 9 shows the optimum conditions for the model reaction.

**TABLE 1:** Optimization of the reaction condition for the synthesis of **6a** as the model reaction^a^.

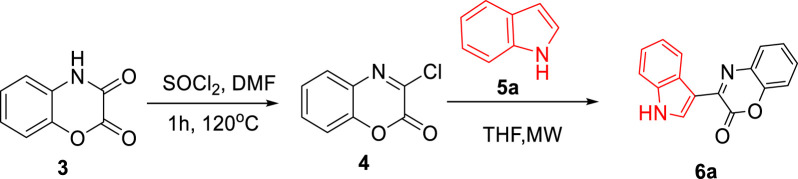				
Entry	Solvent	Condition	Time (min)	Yield (%)
1	CHCl_3_	Reflux	120	50
2	THF	Reflux	120	60
3	CH_3_CN	Reflux	180	30
4	DMF	Reflux	180	10
5	MeOH	Reflux	30	10
^b^6	Cl-CH_2_CH_2_-Cl	Reflux	180	40
7	THF	r.t	180	60
8	THF	Ball-mill	12	70
^c^ **9**	**THF**	**MW**	**7**	**85**

^a^ All reactions were performed using **4a** (0.55 mmol), **5a** (1.1 mmol), and 2 mL of the solvent.

^b^ AlCl_3_.

^c^ Optimized reaction conditions.

**Scheme 2:**
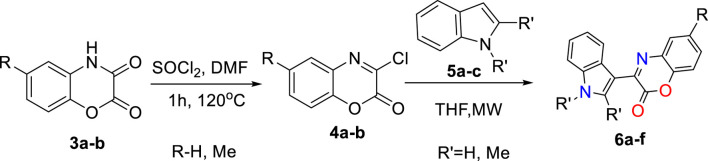
Two-step preparation of 3-aryl-2H-benzo[b][1,4]oxazin-2-one scaffolds 6a-f via S_N_Ar reactions.

As shown in Scheme 2, by treating 1,4-benzoxazinedione (**3a-b)** with Vilsmeier–Haack reagent, a series of 3-chloro-1,4-benzoxazin-2-one derivatives (**4a-b)** were synthesized containing an electrophile center which can undergo the S_N_Ar reaction in the presence of indoles 5a-c as the nucleophile to give the desired 3-aryl-2H-benzo[b][1,4]oxazin-2-ones (**6a-f)**.

**FIGURE 2:**
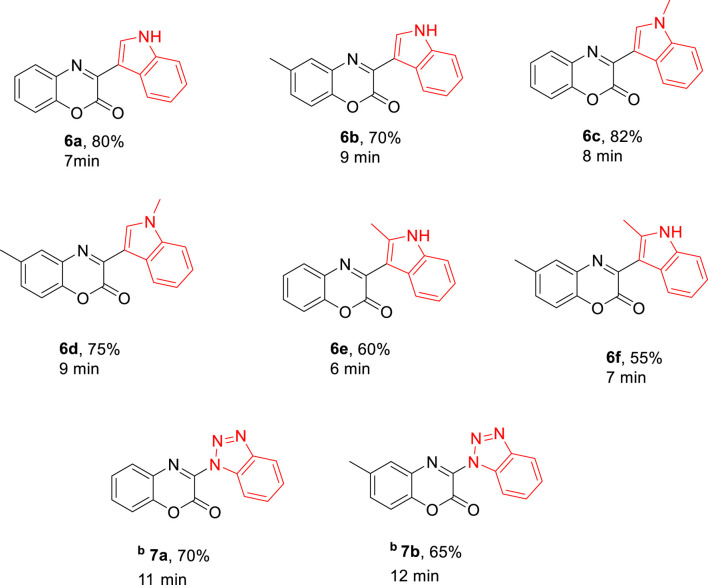
Preparation of compounds **6a-f** ^a^ (^a^All reactions were performed using imide-chloride **4a-b** (0/55 mmol) and indole **5a-c** (1/1 mmol) in THF (2 mL) under microwave 300 W. ^b^Benzotriazole (0/55 mmol) was used instead of indole **5a-c**).

For the synthesis of 3-aryl-2*H*-benzo[b][1,4]oxazin-2-ones derivatives, 1,4-benzoxazinediones (**3a-b**) were primarily synthesized by cyclization reactions of 2-aminophenol **(1a-b)** with oxalyl chloride. Subsequently, these 1,4-benzoxazinediones were treated with Vilsmeier–Haack reagent to obtain 3-chloro-1,4-benzoxazin-2-one derivatives (**4a-b)**. The desired natural products cephalandole A and derivatives **6a-f** were obtained under the optimal conditions mentioned in Figure 2. A comparison of the results shows that bearing an -CH_3_ group on the benzene ring in **4b** produced lower yields of products than those analogs without the -CH_3_ substituent, so the presence of electron-donating groups on benzoxazine led to a decrease in product yields. N-methylindole gave the product with a higher yield than indole and 2-methyl indole.

It would be especially mentioned that benzotriazole is also a suitable substrate for this transformation, and corresponding desired products (7a, 7b) were isolated in 70% and 65% yield, respectively. Some products were characterized by FT-IR, ^1^H NMR, and ^13^C NMR spectroscopy. Cephalandole A and its derivatives were formed through aromatic substitution of the nucleophile in (**4a-b**) due to the high electrophilicity at the C2 position of the dione. Previous studies have shown that if the nucleophilic moiety is strong enough, it will attack the carbonyl group. It can be concluded that the nucleophile plays an important role in the reaction mechanism.

### 2.2 *In silico* studies

Given the notable biological importance of benzoxazine and indole compounds, which have been previously documented as effective antimicrobial agents against *Staphylococcus aureus*, including methicillin-resistant *Staphylococcus aureus* (MRSA), as well as *Mycobacterium tuberculosis*, we aimed to investigate their interactions with the protein structures of RelMtb from *M. tuberculosis* (PDB 5XNX) and RelSeq from *Streptococcus dysgalactiae* subsp. equisimilis (PDB 1VJ7). RelMtb is an integral component of the stringent response pathway, a highly conserved bacterial stress response mechanism enabling bacteria to acclimate to conditions of nutrient scarcity and other environmental stressors. Its pivotal role encompasses the synthesis and degradation of the signaling molecule (p)ppGpp. Consequently, pursuing antibacterial agents directed at RelMtb emerges as a promising and strategically sound initiative for targeting *Mycobacterium tuberculosis* (Vercruysse et al., 2011; Irving and Corrigan, 2018). Consequently, we employed a library of compounds to investigate their potential binding interactions with RelMtb or its active sites, using molecular docking methods. The molecular docking analysis revealed that compounds **6a-7b** exhibited favorable binding affinities within the active site of RelMtb, with docking scores ranging from −8.866 kcal/mol to −7.067 kcal/mol (Table 2). Our *in silico* study unequivocally demonstrated the effective involvement of both the heterocyclic ring benzoxazin-2-one and indole in interactions with the RelMtb crystal structure, which was in an unbound state concerning the substrate. Furthermore, in most instances, interactions with His177 were observed.

**TABLE 2:** Results of molecular docking study of **6a-7b** against the *Mycobacterium tuberculosis* binding site.

Entry	Binding energy	Moiety of compound	Residue	Type of interaction
**6a**	−7.067	Indole Indole	His177 His177	Pi–pi stacking Pi–pi stacking
**6b**	−8.866	C=O of benzooxazin-2-one NH of indole Indole Indole	Trp345 Arg346 His177 Tyr306	H-bound H-bound Pi–pi stacking Pi–pi stacking
**6c**	−7.811	C=O of benzooxazin-2-one	His344	H-bound
**6d**	−7.958	C=O of benzooxazin-2-one Indole	His344 Typ306	H-bound Pi–pi stacking
**6e**	−8.267	C=O of benzooxazin-2-one NH of indole Indole Indole	Gly307 His177 His177 Tyr306	H-bound H-bound Pi–pi stacking Pi–pi stacking
**6f**	−7.134	Benzooxazin-2-one Indole Indole Indole Indole	His344 Ala350 Lys127 Trp345 Trp345	Pi–pi stacking H-bound Pi-cation Pi stacking Pi stacking
**7a**	−6.342	Indole	His177	Pi–pi stacking
**7b**	−8.150	Benzooxazin-2-one C=O of benzooxazin-2-one	Tyr306 His344	Pi–pi stacking H-bound

Next, we assessed the impact of the compounds on the RelSeq structure within the synthetic domain (SYN), which was complexed with a GDP (guanosine diphosphate) substrate, to investigate their binding affinity to (p)ppGpp synthetase in a catalytically active state. This analysis observed weaker binding interactions within the RelSeq active site, with docking scores ranging from −6.639 to −4.061 kcal/mol. Notably, both indole and benzoxazin-2-one demonstrated favorable interactions with the binding site. In most instances, interactions with the Tyr308 residue, which was crucial for RelMtb (p)ppGpp synthesis, were also observed (Table 3).

**TABLE 3:** Results of molecular docking study of **6a-7b** against the *Streptococcus dysgalactiae* binding site.

Entry	Binding energy	Moiety of compound	Residue	Type of interaction
**6e**	−4.161	C=O of benzooxazin-2-one Benzooxazin-2-one Benzooxazin-2-one Benzooxazin-2-one Indole Indole	Arg241 Arg241 Arg269 Trp185 Arg241 Lys243	H-bound Pi-cation Pi-cation Pi–pi stacking Pi-cation Pi-cation
**6a**	−4.692	O of benzooxazin-2-one C=O of benzooxazin-2-one Benzooxazin-2-one Indole	Trp185 Arg241 Arg327 Lys243	H-bound H-bound Pi-cation Pi-cation
**6b**	−6.639	C=O of benzooxazin-2-one C=O of benzooxazin-2-one NH of benzooxazin-2-one Benzooxazin-2-one NH of indole Indole Indole Indole	Lys184 Trp185 Gln325 Lys304 Ala335 Arg327 Tyr308 Tyr308	H-bound H-bound H-bound Pi-cation H-bound Pi-cation Pi–pi stacking Pi–pi stacking
**6c**	−5.099	Benzo [d][1,2,3]triazole Indole	Arg269 Arg241	H-bound Pi-cation
**6d**	−4.280	Benzooxazin-2-one Indole Indole	Trp185 Trp185 His176	Pi–pi stacking Pi–pi stacking Pi–pi stacking
**6e**	−4.615	Benzooxazin-2-one Benzooxazin-2-one Benzooxazin-2-one Indole	Arg269 Arg327 Tyr308 Glu323	Salt bridge Pi-cation Pi–pi stacking H-bound
**6f**	−4.889	NH of benzoxazine Benzoxazine Indole	Arg269 Arg327 Tyr308 Glu323	Salt bridge Pi-cation Pi–pi stacking H-bound
**7a**	−4.482	Benzo [d][1,2,3]triazole Benzo [d][1,2,3]triazole	Arg241 Arg269	H-bound H-bound
**7b**	−4.061	Benzo [d][1,2,3]triazole Benzo [d][1,2,3]triazole Benzooxazin-2-one	Arg269 Typ308 Lys243	H-bound Pi–pi stacking H-bound

**FIGURE 3:**
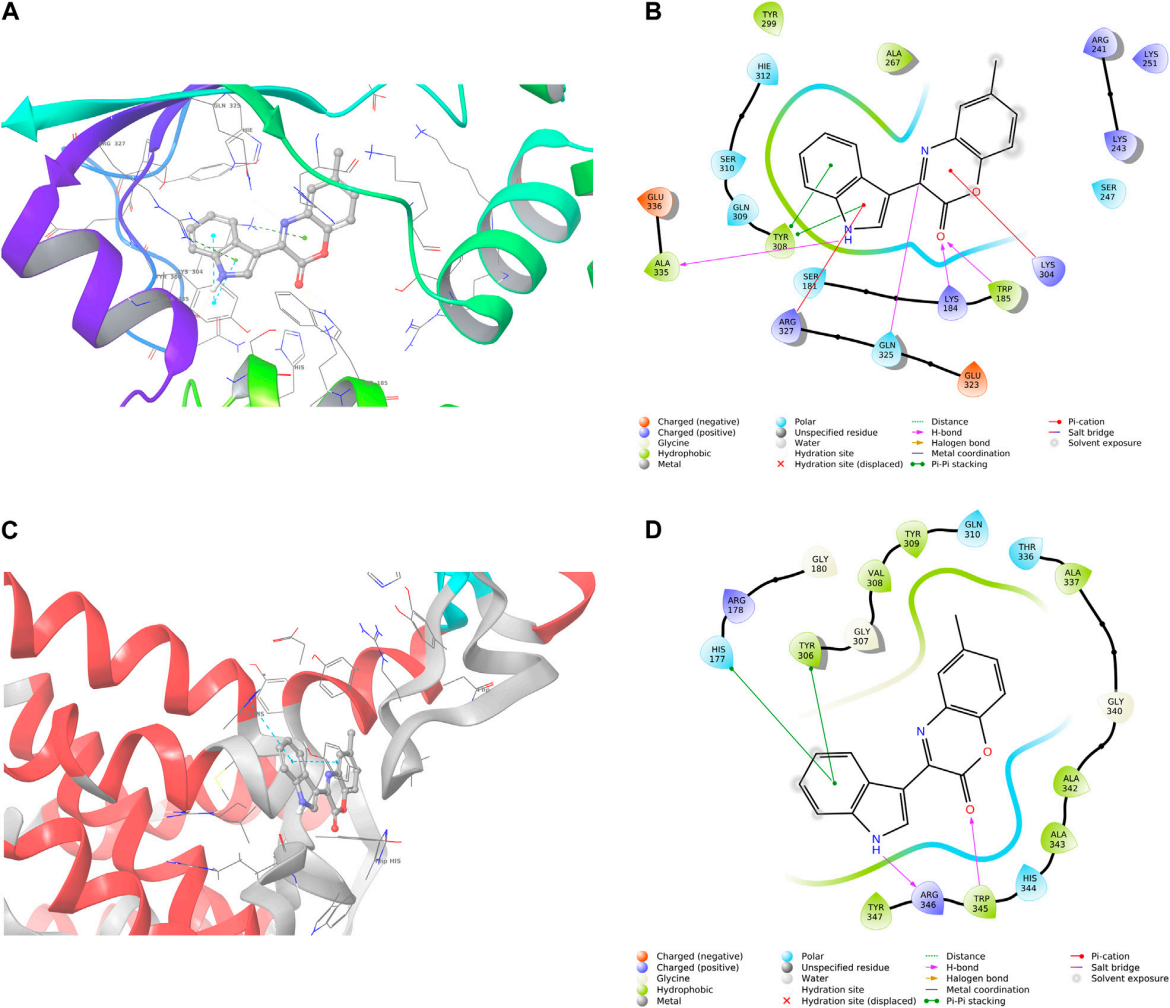
Three-dimensional and two-dimensional interaction patterns of compound **6b** within the binding sites of RelMtb **(A, B)** and RelSeq **(C, D)**. **(A)** Three-dimensional interaction of **6b** in RelMtb of the 5XNX binding site. **(B**) Two-dimensional interaction of **6b** in RelMtb of the 5XNX binding site. **(C**) Three-dimensional interaction of **6b** in the RelSeq structure of the 1vj7 binding site. **(D)** Two-dimensional interaction of **6b** in the RelSeq structure of the 1vj7 binding site.

The binding pose and interaction of compound **6b** were characterized as potent antibacterial agents against both RelMtb in *Streptococcus dysgalactiae* and the RelSeq structure of *Streptococcus dysgalactiae* illustrated in Figure 3, and according to the obtained results, this compound holds promise for further optimization and development.

### 2.3 Reaction mechanism

**FIGURE 4:**
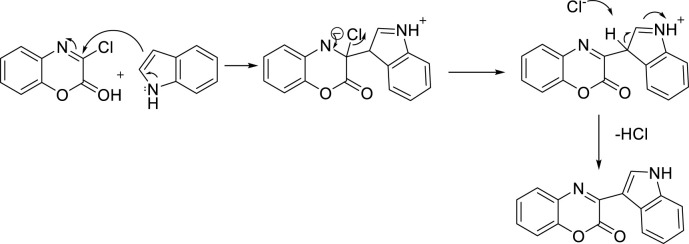
Suggested pathway of the reaction.

The suggested reaction pathway is given below in Figure 4. According to the proposed route, the starting point of the reaction includes the nucleophilic addition of carbon number 3 position of indole to the electrophilic center of 1,4-benzoxazine, and the desired product is obtained after elimination of an HCl molecule.

## 3 Conclusion

In summary, a new environmentally efficient method has been developed to directly couple 3-chloro-1,4-benzoxazin-2-one with indoles, enabling the synthesis of diverse functionalized benzoxazines This process, conducted under microwave irradiation, is characterized by its simplicity, safety, and metal-free nature, yielding high-purity products in significant quantities. Additionally, this method facilitates access to the natural product cephalandole A. Furthermore, *in silico* approaches demonstrated that 3-aryl-2*H*-benzo[b][1,4]oxazin-2-ones have potential antimicrobial activity against *Mycobacterium tuberculosis* and *Streptococcus dysgalactiae*. This sustainable, eco-friendly, and expeditious methodology can be considered an alternative means for development of novel biologically active agents.

## 4 Method and materials

### 4.1 General procedure of the synthesis of 1,4-benzoxaxinedione 3a

To a solution of oxalyl chloride (4.2 mmol) in o-dichlorobenzene (3 mL), 2-aminophenol (3 mmol) was added at room temperature, and the reaction mixture was stirred at 120°C for 3 h. As the reaction progressed, HCl vapors formed, and a slurry suspension was produced. The progress of the reaction was monitored by thin-layer chromatography (TLC). Once the reaction was complete, the white precipitate was filtered and first washed with o-dichlorobenzene and then with toluene. The product was obtained as a white powder (90%).

#### 4.1.1 General procedure of the synthesis of 3-chloro-1,4-benzoxazine-2-one (4a-b)

To a solution of (2.45 mmol) **3a-b** in dry toluene (5 mL) after adding (0.1 mL) DMF, 0.2 mL (2.75 mmol) thionyl chloride was added dropwise at the boiling point of the solvent. The solution was stirred at 120°C for 1 h. The reaction mixture was evaporated under a vacuum. The residue was purified by recrystallization using benzene/petroleum ether (1:2) to afford the desired product **4** as a colorless crystal (70%–80%).

#### 4.1.2 The general procedure of the synthesis of cephalandole A and analogous (6a-f)

To (0.55 mmol) 3-chloro-1,4-benzoxazine-2-one (**4a-b)** and (1.1 mmol) indole (**5a-c)** was added (2 mL) THF. The mixture was placed in a microwave synthesizer with a power of 300 w and a temperature of 60°C for 6–12 min. The progress of the reaction was monitored by thin-layer chromatography (EtOAc/n-hexane 3:20). After the completion of the reaction, the solvent was removed, and the crude product was recrystallized twice from ethanol to afford the pure cephalandole A and its analogous **6a-f** in good yields (55%–82%).

#### 4.1.3 The general procedure of the synthesis of 7a-b

To (0.55 mmol) **4a-b** and (1.1 mmol) benzotriazole was added (2 mL) THF. The mixture was placed in a microwave with a power of 300 w and a temperature of 60°C for 11–12 min. Then, the solvent was removed, and the desired product **7a-b** was precipitated. The crude product was washed with ethanol and dried *in vacuo* (65%–70%).

#### 4.1.2 2*H*-benzo[b][1,4]oxazine-2,3(4H)-dione (3a)

Yield 90%; white powder; mp: 275°C–276°C, IR (KBr, cm^−1^) v = 1700, 1775; ^1^H-NMR (499 MHz, DMSO-*d*_*6*_) δ 11.89 (s, 1H), 7.25 (d, J = 8.0 Hz, 1H), 7.20 (t, J = 7.6 Hz, 1H), 7.11 (t, J = 8.2 Hz, 2H); ^13^C NMR (126 MHz, DMSO-d_6_) δ 153.99, 150.83, 140.56, 125.57, 124.84, 123.11, 116.28, and 115.67.

#### 4.1.3 6-methyl-2*H*-benzo[b][1,4]oxazine-2,3(4H)-dione (3b)

Yield 90%; white powder; mp: 236°C–239°C, ^1^H-NMR (400 MHz, DMSO-*d*_6_) δ 11.87 (s, 1H), 7.12 (d, *J* = 8.2 Hz, 1H), 6.97–6.77 (m, 2H), 2.28 (s, 3H); ^13^C NMR (101 MHz, DMSO-*d*_*6*_) δ 154.47, 151.30, 138.97, 134.67, 125.59, 124.11, 116.45, 116.07, and 20.89.

#### 4.1.4 3-chloro-1,4-benzoxazin-2-one (4a)

Yield 70%; colorless crystal; mp: 138°C–140°C, IR (KBr, cm^−1^) v = 1760, ^1^H-NMR (499 MHz, acetone-*d*_*6*_) δ 7.74 (dd, J = 8.0, 1.7 Hz, 1H), 7.68 (td, J = 7.9, 1.7 Hz, 1H), 7.50 (td, J = 7.7, 1.3 Hz, 1H), 7.44 (dd, J = 8.2, 1.4 Hz, 1H); ^13^C-NMR (126 MHz, acetone-d_6_) δ 149/35, 146/83, 145/80, 131/71, 130/64, 128/35, 125/77, and 116/35.

#### 4.1.5 3-chlor-6-methyl-2-oxo-1,4-benzoxazin (4b)

Yield 80%; colorless crystal; mp: 138°C–139°C, ^1^H-NMR (400 MHz, acetone-*d*_*6*_) δ 7.15 (d, J = 8.3 Hz, 1H), 7.05 (d, J = 2.0 Hz, 1H), 7.00 (dd, J = 8.3, 2.0 Hz, 1H), 2.35 (s, 3H); ^13^C-NMR (101 MHz, acetone) δ 153.53, 150.28, 138.83, 135.02, 124.98, 124.16, 116.23, 115.76, and 19.95.

#### 4.1.6 3-(1*H*-indol-3-yl)-2*H*-benzo[b][1,4]oxazin-2-one (cephalandole A) (6a): yield 80%; bright yellow.

Amorphous powder; mp: 237°C–238°C, IR (KBr, cm^−1^) v = 3,301, 1718, 1606,1533,1,431 cm^-1^, ^1^H-NMR (499 MHz, DMSO-*d*_*6*_) δ 11.98 (s, 1H), 8.79–8.73 (m, 1H), 8.70 (d, J = 3.0 Hz, 1H), 7.89–7.84 (m, 1H), 7.61–7.50 (m, 1H), 7.50–7.45 (m, 1H), 7.45–7.39 (m, 2H), 7.31–7.23 (m, 2H); ^13^C-NMR (126 MHz, DMSO-d_6_) δ 152.51, 148.37, 145.33, 137.05, 134.21, 132.41, 129.13, 128.17, 126.44, 125.79, 123.51, 123.29, 121.97, 116.37, 112.66, and 111.04, anal. Calcd. For C_16_H_10_N_2_O_2_ (C, 73.27; H, 3.84; N, 10.68); found: (C, 73.54; H, 3.78; N, 10.57).

#### 4.1.7 3-(1*H*-indol-3-yl)-6-methyl-2*H*-benzo[b][1,4]oxazin-2-one (6b)

Yield 70%; brown to yellow powder; mp: 257°C–259°C, ^1^H-NMR (400 MHz, DMSO-*d*_*6*_) δ 11.98 (s, 1H), 8.77 (dd, J = 7.3, 2.1 Hz, 1H), 8.69 (d, J = 3.0 Hz, 1H), 7.67 (d, J = 1.7 Hz, 1H), 7.54 (dd, J = 7.3, 1.8 Hz, 1H), 7.40–7.09 (m, 4H), 2.42 (s, 3H); ^13^C-NMR (101 MHz, DMSO) δ 152.64, 148.22, 143.24, 137.03, 135.16, 134.08, 132.07, 129.91, 128.05, 126.45, 123.48, 123.32, 121.91, 115.95, 112.65, 111.09, and 20.81, anal. Calcd. For C_17_H_12_N_2_O_2_ (C, 73.90; H, 4.38; N, 10.14); found: (C, 73.78; H, 4.26; N, 10.09).

#### 4.1.8 3-(1-methyl-1*H*-indol-3-yl)-2*H*-benzo[b][1,4]oxazin-2-one (6c)

Yield 82%; bright yellow crystal; mp: 203°C–206°C, ^1^H-NMR (400 MHz, DMSO-*d*_6_) δ 8.81–8.75 (m, 1H), 8.72 (s, 1H), 7.85 (dd, *J* = 7.6, 1.7 Hz, 1H), 7.59 (dd, *J* = 7.3, 1.7 Hz, 1H), 7.52–7.27 (m, 5H), 3.94 (s, 3H); ^13^C-NMR (101 MHz, DMSO-*d*_*6*_) δ 152.47, 148.02, 145.30, 137.87, 137.64, 132.41, 129.10, 128.14, 126.90, 125.79, 123.58, 123.44, 122.31, 116.35, 111.04, 109.98, and 33.63, anal. Calcd. For C_17_H_12_N_2_O_2_ (C, 73.90; H, 4.38; N, and 10.14); found: (C, 74.11; H, 4.42; N, 10.09).

#### 4.1.9 6-methyl-3-(1-methyl-1*H*-indol-3-yl)-2*H*-benzo[b][1,4]oxazin-2-one (6d)

Yield 75%; yellow solid; mp: 225°C–227°C, IR (KBr, cm^−1^) v = 2,908, 1725, 1527,744, ^1^H-NMR (400 MHz, DMSO-*d*_6_) δ 8.82–8.75 (m, 1H), 8.71 (s, 1H), 7.68 (s, 1H), 7.63–7.56 (m, 1H), 7.39–7.25 (m, 4H), 3.94 (s, 3H), 2.43 (s, 3H), ^13^C-NMR (101 MHz, DMSO-*d*_*6*_) δ 152.63, 147.92, 143.25, 137.76, 137.64, 135.19, 132.10, 129.92, 128.04, 126.92, 123.57, 123.45, 122.27, 115.97, 111.05, 110.03, 33.63, and 20.81, anal. Calcd. For C_18_H_14_N_2_O_2_ (C, 74.47; H, 4.86; N, 9.65); found: (C, 74.18; H, 4.85; N, 9.68).

#### 4.1.10 3-(2-methyl-1*H*-indol-3-yl)-2*H*-benzo[b][1,4]oxazin-2-one (6e)

Yield 60%; red to brown crystal; mp: 184°C–185°C, IR (KBr, cm^−1^) v = 3,323, 1730, 1453,733, ^1^H NMR (499 MHz, DMSO-*d*_*6*_) δ 11.69 (s, 1H), 7.87 (d, J = 7.9 Hz, 1H), 7.77 (dt, J = 7.9, 1.5 Hz, 1H), 7.55–7.47 (m, 1H), 7.47–7.38 (m, 2H), 7.36 (d, J = 7.9 Hz, 1H), 7.15–7.03 (m, 2H), 2.59 (s,3H), anal. Calcd. For C_17_H_12_N_2_O_2_ (C, 73.90; H, 4.38; N, 10.14); found: (C, 73.75; H, 4.37; N, 10.11).

#### 4.1.11 6-methyl-3-(2-methyl-1*H*-indol-3-yl)-2*H*-benzo[b][1,4]oxazine (6f)

Yield 55%; red to brown crystal; mp: 203°C–204°C, ^1^H NMR (400 MHz, DMSO-*d*_*6*_) δ 11.71 (s, 1H), 7.92–7.85 (m, 1H), 7.61–7.56 (m, 1H), 7.38 (d, J = 7.9 Hz, 1H), 7.30 (d, J = 1.3 Hz, 2H), 7.17–7.03 (m, 2H), 2.60 (s, 3H), 2.41 (s, 3H), ^13^C NMR (101 MHz, DMSO-*d*_*6*_) δ 152.88, 150.52, 144.02, 141.37, 135.59, 135.03, 131.99, 130.49, 128.23, 128.00, 121.80, 121.45, 120.46, 115.95, 111.25, 108.79, 20.76, and 14.86, anal. Calcd. For C_18_H_16_N_2_O_2_ (78.24; H, 5.84; N, 10.14); found: (C, 77.94; H, 5.79; N, 10.10).

#### 4.1.12 3-(1*H*-benzo[d][1,2,3]triazol-1-yl)-2H-benzo[b][1,4]oxazin-2-one (7a)

Yield 70%; yellowish crystal; mp: 191°C–192°C, IR (KBr, cm^−1^) v = 1751, 1,182, 994, 749, ^1^H-NMR (400 MHz, DMSO-*d*_*6*_) δ 8.24 (t, J = 8.7 Hz, 2H), 7.93 (dd, J = 7.9, 1.5 Hz, 1H), 7.73 (ddd, J = 22.5, 8.7, 7.1, 1.3 Hz, 2H), 7.59 (ddt, J = 6.9, 3.8, 1.8 Hz, 2H), 7.53 (td, J = 7.6, 1.4 Hz, 1H), ^13^C-NMR (101 MHz, DMSO-*d*_*6*_) δ 148.99, 147.04, 145.41, 142.25, 132.64, 132.14, 130.05, 129.72, 129.22, 126.27, 126.07, 120.06, 116.76, and 114.80, anal. Calcd. For C_14_H_18_N_2_O_2_ (C, 63.64; H, 3.05; N, 21.20); found: (C, 63.46; H, 3.04; N, 21.42).

#### 4.1.13 3-(1*H*-benzo[d][1,2,3]triazol-1-yl)-6-methyl-2*H*-benzo[b][1,4]oxazin-2-one (7b)

Yield 65%; deep yellow crystal; mp: 194°C–195°C, ^1^H NMR (400 MHz, DMSO-*d*_*6*_) δ 8.28–8.18 (m, 2H), 7.79–7.70 (m, 2H), 7.59 (ddd, J = 8.2, 7.0, 1.1 Hz, 1H), 7.54–7.43 (m, 2H), 2.44 (s, 3H), ^13^C NMR (101 MHz, DMSO-*d*_*6*_) δ 149.08, 145.39, 144.99, 142.12, 135.84, 132.89, 132.63, 129.72, 129.66, 128.95, 126.04, 120.04, 116.36, 114.78, and 20.70, anal. Calcd. For C_15_H_10_N_4_O_2_ (C, 64.74; H, 3.62; N, 20.13); found: (C, 64.9; H, 3.88; N, 20.05).

### 4.2 Molecular docking study

Molecular docking analyses were conducted using the Schrödinger Maestro software [93]. The analysis utilized the protein structures of RelMtb from *M. tuberculosis* (PDB: https://www.rcsb.org/structure/5xnx) and RelSeq from *Streptococcus dysgalactiae* subsp. equisimilis (PDB: https://www.rcsb.org/structure/1vj7). These structures were initially optimized and minimized with the Protein Preparation Wizard and the OPLS3 force field. Additionally, all derivatives’ structures were optimized using LigPrep. The molecular docking was executed in the IFD mode, with the ligand considered flexible and employing the OPLS-2005 force field, while maintaining default settings for other parameters. A grid for IFD calculation was generated based on the binding site. Up to five poses with receptor and ligand van der Waals radii of 0.7 and 0.5, respectively, were considered. Residues within a 15 Å radius of the crystallographic ligands at the active site underwent refinement, followed by side-chain optimization. Structures with a prime energy exceeding 30 kcal/mol were eliminated.

## Data Availability

The datasets generated and/or analyzed during the current study are available in the Worldwide ProteinData Bank with PDB ID of 5XNX (https://www.rcsb.org/structure/5XNX) and 1vj7 (https://www.rcsb.org/structure/1vj7) repository.
