# Supplementary figures and images for: Sustainable synthesis of antibacterial 3-aryl-2H-benzo[b,1,4]oxazin-2-ones via SNAr Csp2–Csp2 coupling
Source: Front Chem. 2024 Nov 25;12:1472342. doi: 10.3389/fchem.2024.1472342 (PMC11625556; doi:10.3389/fchem.2024.1472342)

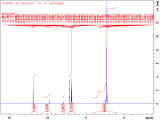

Supplement: Supplementary file 1 [file DataSheet2.zip › NMR FID/3b/6586/pdata/1/thumb.png]

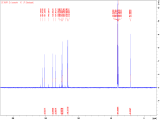

Supplement: Supplementary file 1 [file DataSheet2.zip › NMR FID/3b/6587/pdata/1/thumb.png]

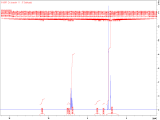

Supplement: Supplementary file 1 [file DataSheet2.zip › NMR FID/4b/6584/pdata/1/thumb.png]

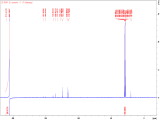

Supplement: Supplementary file 1 [file DataSheet2.zip › NMR FID/4b/6585/pdata/1/thumb.png]

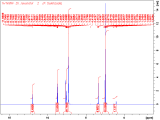

Supplement: Supplementary file 1 [file DataSheet2.zip › NMR FID/6b/6590/pdata/1/thumb.png]

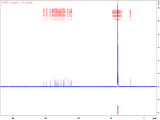

Supplement: Supplementary file 1 [file DataSheet2.zip › NMR FID/6b/6591/pdata/1/thumb.png]
