# Supplementary material for: Sustainable synthesis of antibacterial 3-aryl-2H-benzo[b,1,4]oxazin-2-ones via SNAr Csp2–Csp2 coupling
Source: Front Chem. 2024 Nov 25;12:1472342. doi: 10.3389/fchem.2024.1472342 (PMC11625556; doi:10.3389/fchem.2024.1472342)
Supplement: Supplementary file 2 [file DataSheet1.docx]

**Supporting Information**

**Sustainable Synthesis of Antibacterial 3-Aryl-2*H*-Benzo[b,1,4]oxazin-2-ones *via* S_N_Ar Csp^2^−Csp^2^ Coupling**

******

**Figure S1: IR of compound 3a (KBr)**

******

**Figure S2: ^13^C-NMR of compound 3a (126 MHz, DMSO-d_6_)**

******

**Figure S3: ^1^H-NMR of compound 3a (499 MHz, DMSO-d_6_)**

**Figure S4: IR of compound 3b (KBr)**

**Figure S5: ^13^C-NMR of compound 3b (101 MHz, DMSO-d_6_)**

**Figure S6: ^1^H-NMR of compound 3b (400 Hz, DMSO-d_6_)**

**Figure S7:IR of compound 4a (KBr)**

**Figure S8:^13^C-NMR of compound 4a (126 MHz,Acetone-d_6_)**

**Figure S9: ^1^H-NMR of compound 4a (499 MHz, Acetone-d_6_)**

**Figure S10: ^13^C-NMR of compound 4b (101 MHz, Acetone-d_6_)**

**Figure S11:^1^H-NMR of compound 4b (400 MHz, Acetone-d_6_)**

**Figure S12:IR of compound 6a (KBr)**

**Figure S13:^13^C-NMR of compound 6a (126 MHz, DMSO-d_6_)**

**Figure S14:^1^H-NMR of compound 6a (499 MHz, DMSO-d_6_)**

******

**Figure S15:^13^C-NMR of compound 6b (101 MHz, DMSO-d_6_)**

******

**Figure S16:^1^H-NMR of compound 6b (400 MHZ, DMSO-d_6_)**

******

**Figure S17:IR of compound 6c (KBr)**

******

**Figure S18:^13^C-NMR of compound 6c (101 MHz, DMSO-d_6_)**

******

**Figure S19:^1^H-NMR of compound 6c (400 MHz, DMSO-d_6_)**

******

**Figure S20:IR of compound 6d (KBr)**

******

**Figure S21:^13^C-NMR of compound 6d (101 MHz, DMSO-d_6_)**

******

**Figure S22:^1^H-NMR of compound 6d (400 MHz, DMSO-d_6_)**

******

**Figure S23:IR of compound 6e (KBr)**

******

**Figure S24:^13^C-NMR of compound 6e (126 MHz, DMSO-d_6_)**

******

**Figure S25:^1^H-NMR of compound 6e (499 MHz, DMSO-d_6_)**

**Figure S26:IR of compound 6f (KBr)**

**Figure S27:^13^C-NMR of compound 6f (101 MHz, DMSO-d_6_)**

**Figure S28:^1^H-NMR of compound 6f (400 MHz, DMSO-d_6_)**

**Figure S29:IR of compound 7a (KBr)**

**Figure S30:^13^C-NMR of compound 7a (101MHZ, DMSO-d_6_)**

**Figure S31:^1^H-NMR of compound 7a (400MHz, DMSO-d_6_)**

**Figure S32:^13^C-NMR of compound 7b (101 MHz, DMSO-d_6_)**

**Figure S33:^13^C-NMR of compound 7b (400 MHz, DMSO-d_6_)**
