# Supplementary material for: Sustainable synthesis of antibacterial 3-aryl-2H-benzo[b,1,4]oxazin-2-ones via SNAr Csp2–Csp2 coupling
Source: Front Chem. 2024 Nov 25;12:1472342. doi: 10.3389/fchem.2024.1472342 (PMC11625556; doi:10.3389/fchem.2024.1472342)
Supplement: Supplementary file 1 [file DataSheet2.zip › NMR FID/6f/CNMR.pdf]

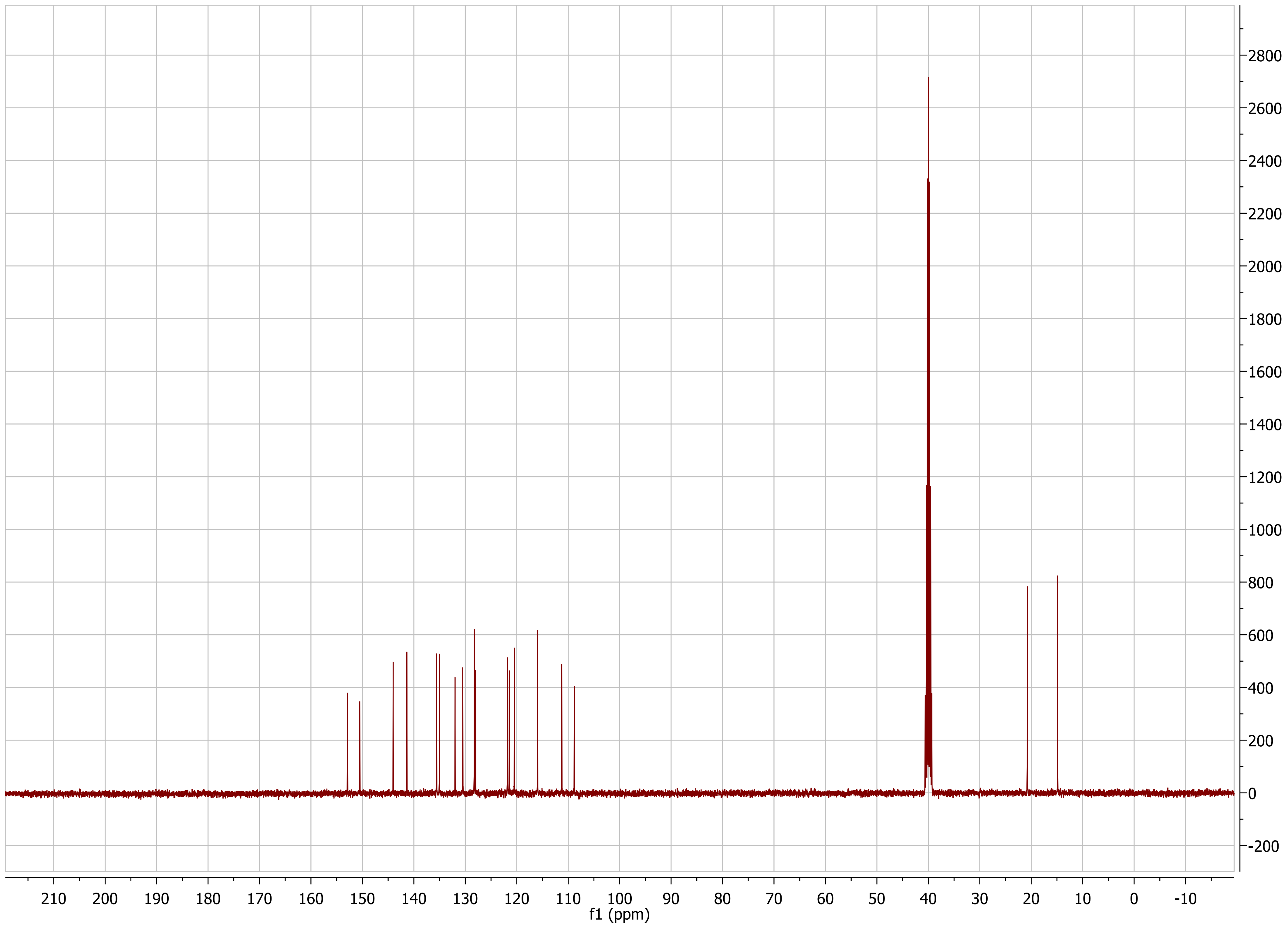

210
200
190
180
170
160
150
140
130
120
110
100
90
80
70
60
50
40
30
20
10
0
-10
f1 (ppm)
2800
2600
2400
2200
2000
1800
1600
1400
1200
1000
800
600
400
200
0
-200
