# Supplementary material for: Sustainable synthesis of antibacterial 3-aryl-2H-benzo[b,1,4]oxazin-2-ones via SNAr Csp2–Csp2 coupling
Source: Front Chem. 2024 Nov 25;12:1472342. doi: 10.3389/fchem.2024.1472342 (PMC11625556; doi:10.3389/fchem.2024.1472342)
Supplement: Supplementary file 1 [file DataSheet2.zip › NMR FID/6f/HNMR.pdf]

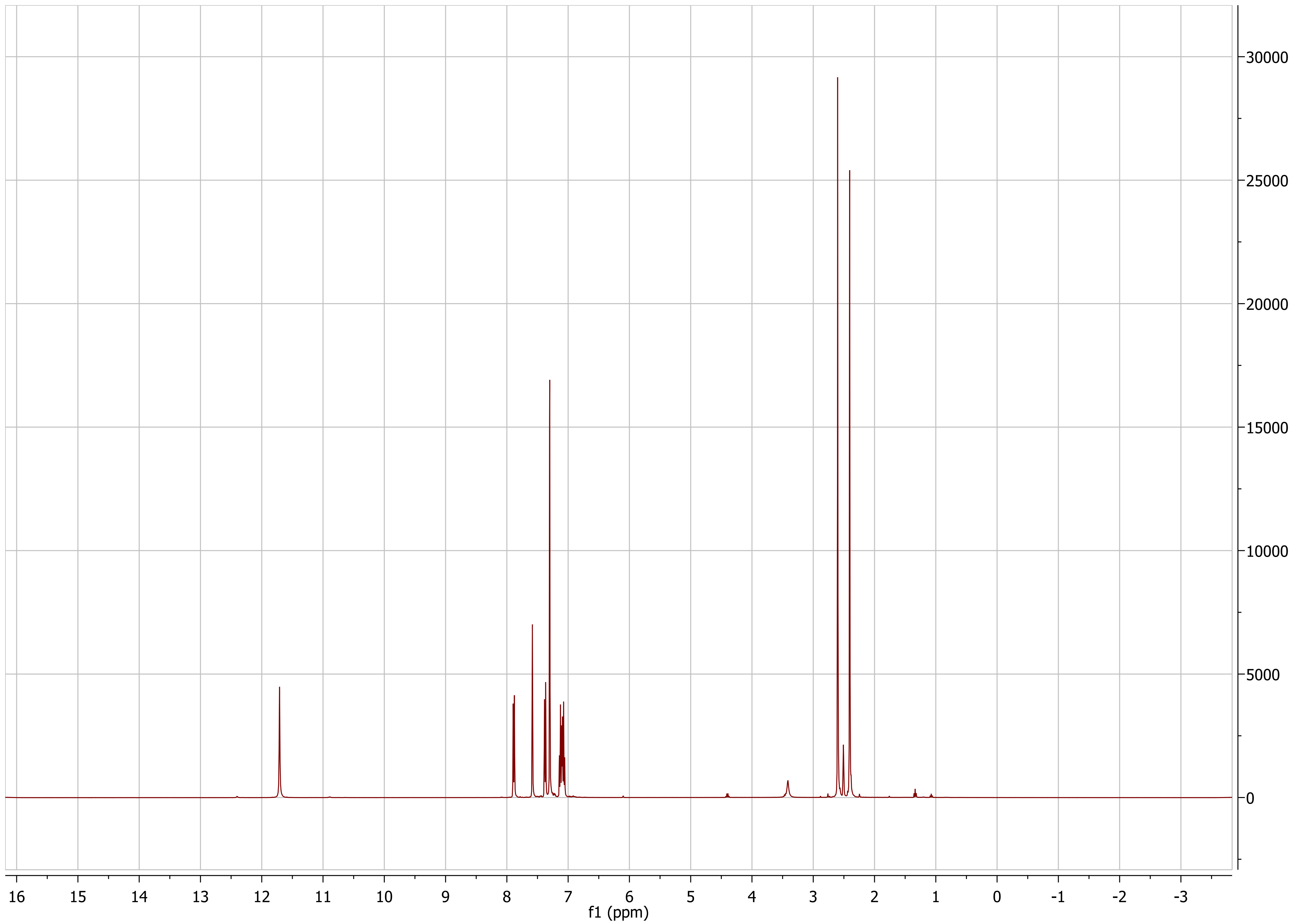

30000
25000
20000
15000
10000
5000
0
16
15
14
13
12
11
10
9
8
7
6
5
4
3
2
1
0
-1
-2
-3
f1 (ppm)
